# Plant glyco-biotechnology on the way to synthetic biology

**DOI:** 10.3389/fpls.2014.00523

**Published:** 2014-10-08

**Authors:** Andreas Loos, Herta Steinkellner

**Affiliations:** Department of Applied Genetics and Cell Biology, University of Natural Resources and Life SciencesVienna, Austria

**Keywords:** glycoengineering, plant, glycosyltransferase, CTS, sub-Golgi targeting

## Abstract

Plants are increasingly being used for the production of recombinant proteins. One reason is that plants are highly amenable to glycan engineering processes and allow the production of therapeutic proteins with increased efficacies due to optimized glycosylation profiles. Removal and insertion of glycosylation reactions by knock-out/knock-down approaches and introduction of glycosylation enzymes have paved the way for the humanization of the plant glycosylation pathway. The insertion of heterologous enzymes at exactly the right stage of the existing glycosylation pathway has turned out to be of utmost importance. To enable such precise targeting chimeric enzymes have been constructed. In this short review we will exemplify the importance of correct targeting of glycosyltransferases, we will give an overview of the targeting mechanism of glycosyltransferases, describe chimeric enzymes used in plant *N*-glycosylation engineering and illustrate how plant glycoengineering builds on the tools offered by synthetic biology to construct such chimeric enzymes.

## INTRODUCTION

The medicinal use of proteins and blood products has a long history. Already in the 15th century ailing pope Innocent VIII was reportedly infused with blood from three healthy boys to give him back vigor and strength, however, with a fatal outcome for all four of them (Rivera et al., 2005). The first successful blood transfusion was made in 1665 between dogs (Felts, 2000) and it took over 150 years for the first successful transfusion between humans (Blundell, 1818). Proteins purified from animal or human tissues (growth hormones, insulin, clotting factors, or other blood components) have been used for medicinal purposes since the beginning of the 20th century (e.g., Eibl, 2008; Blizzard, 2012) and with the advent of recombinant protein production possibilities, many of those proteins are now produced recombinantly. The market for pharmaceutical proteins is assumed to currently amount to roughly 150–200 billion US$, and develops strongly with growth rates of ∼10% and more (Walsh, 2010; Elvin et al., 2013; Aggarwal, 2014). Special drivers of this growth are antibodies and antibody related products, but also other types of proteins are selling well, like insulin, vaccines, erythropoietin, etc. (Aggarwal, 2014).

A current major concern in producing biopharmaceuticals is a special type of post-translation modification (PTM), namely *N*-glycosylation. This PTM is found on a large proportion of pharmaceutically relevant proteins (Walsh, 2010) and can influence protein characteristics like folding and assembly, solubility and charge, serum half-life, functionality, etc. (e.g., Varki, 1993; Roth et al., 2010; Solá and Griebenow, 2010). As different cell types attach different glycans, the characteristics of the *N*-glycosylated protein can be strongly affected by the expression host – a fact that should be considered carefully when choosing the production system. For example, bacteria generally do not glycosylate proteins and yeasts attach larger glycan structures than mammals. Insect cells decorate proteins with paucimannosidic *N*-glycans which are normally not present in humans. Plants produce complex type *N-*glycans similar to humans, however, certain non-mammalian epitopes are attached and more complex human-type glycosylation cannot be produced (for reviews on typcial *N-*glycosylation patterns and glycoengineering of different expression hosts see, e.g., Jacobs and Callewaert, 2009; Loos and Steinkellner, 2012). Another concern is glycan microheterogeneity, i.e., attachment of different *N*-glycans to the same *N*-glycosylation site, as homogeneously glycosylated products are required by the regulatory authorities. Thus, research has focused on modifying the glycosylation characteristics of a variety of expression systems to allow homogeneous, human-type *N*-glycosylation (Umana et al., 1999; Yamane-Ohnuki et al., 2004; Schuster et al., 2005; Cox et al., 2006; Li et al., 2006; Strasser et al., 2009; Pandhal and Wright, 2010; Meuris et al., 2014) and resulted in the production of proteins carrying modified glycans and often showing improved *in vivo* functions.

Plants have proven their capability regarding production speed, ease of scale up and to meet quality standards demanded by regulatory agencies for clinical applications (Gleba et al., 2014; Stoger et al., 2014). Also governmental agencies like the Defense Advanced Research Projects Agency [DARPA] (2012) have recognized the advantages of this technology for the quick manufacturing of vaccines, difficult to produce biopharmaceuticals, etc. This has led to massive investments in research, production facilities complying with current quality standards (Defense Advanced Research Projects Agency [DARPA], 2012; www.federalgrants.com, 2012; Stoger et al., 2014) and the first products on the market. Glucocerebrosidase, an enzyme to treat Gaucher’s disease, has been approved by the FDA in 2013 as the first plant-produced, parenterally applied biopharmaceutical (Zimran et al., 2011; van Dussen et al., 2013). Additionally, several plant-made pharmaceuticals have received approval for clinical trials and other plant-produced products are already marketed as research/diagnostic reagent, medical device, cosmetic product etc. (recently reviewed by Gleba et al., 2014; Goodman, 2014; Stoger et al., 2014). Many of these proteins are glycosylated.

In this review we will discuss the approaches taken to engineer the *N*-glycosylation pathway in *Nicotiana benthamiana* and put a strong focus on recently developed and applied semi-synthetic strategies using chimeric glycosyltransferases.

## PLANT GLYCOSYLATION

In plants as in other eukaryotes, the endoplasmic reticulum (ER) and the Golgi apparatus play the central role in protein glycosylation and contain the majority of glycan modifying enzymes (reviewed by, e.g., Helenius and Aebi, 2001). While the ER and its glycan processing repertoire are largely conserved between phyla (and kingdoms), morphology and function of the Golgi differ to some extent (Loos and Steinkellner, 2012; Aebi, 2013). For example, a main function of the plant but not the mammalian Golgi is to provide large amounts of polysaccharides, a fundamental component of the cell wall (Oikawa et al., 2013). Early *N*-glycosylation steps that take place in ER and *cis*-Golgi are virtually identical in higher eukaryotes, while further processing differs (recently reviewd by, e.g., Loos and Steinkellner, 2012; Bosch et al., 2013). This is mainly due to a drastically reduced repertoire of glycosylation enzymes in plants, where a small number of Golgi-located *N*-glycan processing enzymes gives rise to typically two different glycan structures (Castilho and Steinkellner, 2012). By comparison, over 2000 different *N*-glycans have been described on mammalian proteins which arise from several 100 enzymes in the secretory pathway (Campbell and Yarema, 2005; Ohtsubo and Marth, 2006; Varki, 2006). Notwithstanding these differences, the Golgi of higher eukaryotes shares a remarkably high degree of homology, especially with respect to organization, proteome, and *N*-glycosylation capabilities.

Plant proteins typically carry two major *N*-glycans, complex GnGnXF and paucimannosidic MMXF (Strasser et al., 2004a, 2007a; for glycan nomenclature see http://www.proglycan.com/upload/nomen_2007.pdf). These two glycans contain core α1,3-fucose and β1,2-xylose which are plant-specific glyco-epitopes. They are not produced by mammalian cells and up to 50% of humans have been shown to carry substantial amounts of antibodies directed against these epitopes in their blood (Bardor et al., 2003). The abundantly present paucimannosidic structures (MMXF, truncated glycans lacking terminal GlcNAc residues; Dirnberger et al., 2001; Strasser et al., 2007a; Liebminger et al., 2011) are also a plant peculiarity, otherwise only found in insect cells (Altmann et al., 1999, 2001). In some cases plant proteins carry so-called Lewis A epitopes, terminally β1,3-galactosylated and α1,4-fucosylated structures (Fitchette-Laine et al., 1997; Strasser et al., 2007b). The abundance of this epitope differs strongly between species (Fitchette et al., 1999; Wilson et al., 2001) and organs (Strasser et al., 2007b), but seems low in *Arabidopsis* and *Nicotiana* (Fitchette et al., 1999; Strasser et al., 2007b, 2008; Matsuo and Matsumura, 2011). Noteworthy is also the absence of core α1,6-fucosylation in plants, a glycan residue present on the vast majority of proteins produced in mammalian cells. Removal of this residue from immunoglobulin G (IgG) glycans increases functional activities of antibodies due to a higher affinity to the antibody-dependent cell-mediated cytotoxicity (ADCC) inducing IgG receptor FcγRIIIa (Shinkawa et al., 2003; Jefferis, 2009).

The limited glycosylation capacity of plants has turned out to be an advantage for the generation of proteins that need homogeneous glycosylation. For example, IgG antibodies produced in plants carry usually 1–2 different glycan structures (mainly GnGnXF) while the same antibodies produced in Chinese hamster ovary (CHO) cells bear 5–7 structures (Strasser et al., 2008, 2009). For some applications, like testing of functional activities, and according to the demands from regulatory agencies, homogeneous glycosylation is required.

Plants display a remarkable tolerance toward the manipulation of their intrinsic glycan biosynthetic pathways. Elimination of complex glycans, knock-out of plant-specific xylosyl- and fucosyltransferases (XT and FT) or reduction/overproduction of the Lewis A epitope did not lead to any obvious phenotype in *Arabidopsis thaliana* under standard growth conditions (Von Schaewen et al., 1993; Strasser et al., 2004b, 2007b). Also *Lemna minor* and *N. benthamiana*, one of the major plant-based protein production platforms, tolerate a variety of glycoengineering steps without obvious phenotypes or impact on development (Cox et al., 2006; Strasser et al., 2008; Nagels et al., 2011). Only few cases of sensitive reactions to glycosylation changes have been described (Fanata et al., 2013). This general tolerance for glycoengineering was a prerequisite for humanizing the plant *N-*glycosylation pathway. A combination of knock-out/knock-down and knock-in approaches together with transient expression techniques has allowed the removal of potentially immunogenic residues, and the addition of new, human-type glyco-structures. Modular, semi-synthetic constructs assembled on multi-gene vectors enable the efficient manipulation of the glycosylation pathway. These glycoengineering strategies are addressed below.

## PLANT GLYCOENGINEERING

Engineering of plant glycans toward human structures requires two main types of modification: (i) plant-specific reactions have to be eliminated and (ii) reactions taking place in humans but not in plants have to be introduced. Reducing the unwanted plant-specific modifications, i.e., β1,2-xylosylation and core α1,3-fucosylation, has initially been achieved by targeting the recombinant protein to the ER or co-overexpressing glycosylation enzymes competing for the same substrate (e.g., Palacpac et al., 1999; Bakker et al., 2006; Frey et al., 2009; Vézina et al., 2009; Karg et al., 2010; see below). However, as these approaches interfere with the execution of endogenous glycosylation processes and cause the attachment of oligomannosidic or incompletely processed and aberrant glycans they are only of limited use. RNAi approaches targeting the transcript of the unwanted glycosyltransferases or complete knock-outs by T-DNA insertion have proven more successful (Koprivova et al., 2004; Strasser et al., 2004b, 2008; Cox et al., 2006; Sourrouille et al., 2008; Shin et al., 2011; Parsons et al., 2012). Importantly, such plants produce human-type GnGn glycans, which serve as an acceptor substrate for further mammalian modifications and were thus important milestones in the engineering of the plant *N*-glycosylation pathway toward the production of human-type structures.

**FIGURE 1:**
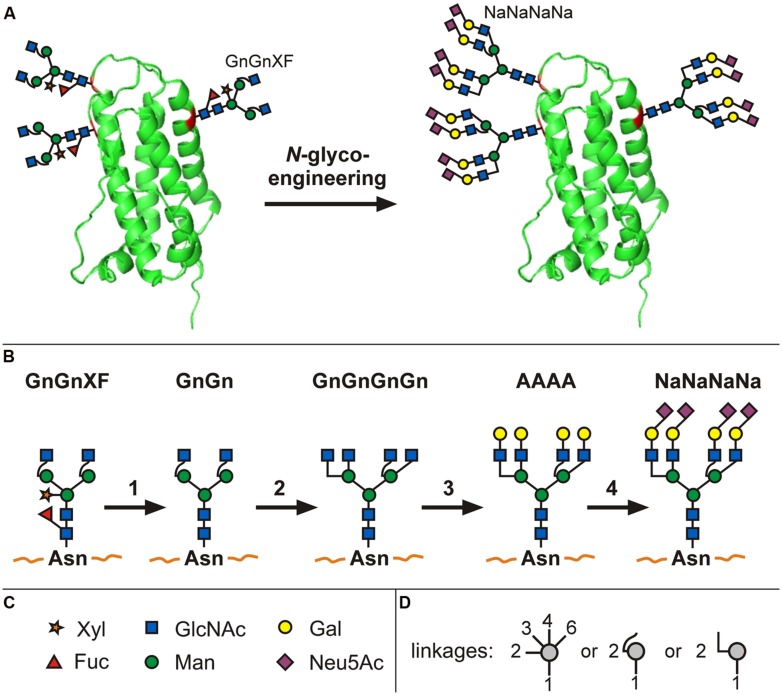
***N-*glycoengineering in plants to produce tetra-sialylated proteins.** Schematic representation of an extensively glycoengineered plant-produced glycoprotein (erythropoietin; **A**). It represents the front of plant glycan engineering and illustrates, in short, the transformation of glycans present in *Nicotiana benthamiana* wild-type plants (GnGnXF) to finally obtain a glycosylation profile present on human serum EPO (NaNaNaNa). For such intensive *N*-glycoengineering, a series of individual steps are necessary (**B**; 1): knock-out or knock-down of plant-specific β1,2-XT and core α1,3-FT, (2) introduction of GnTIV and GnTV responsible for branching, (3) introduction of β1,4-GalT, (4) introduction of sialyltransferase in combination with the biosynthetic pathway to produce activated sialic acid (not shown). Symbols for monosaccharides are given in **(C)**, symbols depicting which monosaccharide atoms are involved in the linkage are given in **(D)**. For detailed description see publications by Castilho et al. (2010, 2011, 2012, 2013). A more detailed explanation of *N*-glycan nomenclature and graphical illustrations can be found at

Consequently, work over the past decade on the controlled expression of mammalian glycosyltransferases has established plant-based systems that synthesize a series of defined human-type glycan structures (Castilho et al., 2012; recently reviewed by Bosch et al., 2013). Recent studies demonstrate how even entire glycosylation-associated biosynthetic pathways can be introduced. Plants do not have the machinery to synthesize the sugar nucleotide precursor CMP-sialic acid (CMP-*N*-acetylneuraminic acid) necessary for sialylation. The simultaneous overexpression of six mammalian genes enabled the *in planta* generation of activated sialic acid, the transfer of the activated sugar nucleotide to the Golgi, the production of terminally galactosylated glycans and the transfer of sialic acid to these terminal galactoses (see **Figures 1A–D**; Castilho et al., 2008, 2010).

*In planta* sialylation of glycans thereby highlights some of the reasons why simple overexpression of a mammalian glycosyltransferase in plants has not always proven successful in generating human-type glycans: acceptor as well as donor substrates need to be present. For example, when Wee et al. (1998) expressed the human α2,6-sialyltransferase in *Arabidopsis*, activity of the enzyme could only be shown after applying donor and acceptor substrates in *trans*, as plants lack both. The achievements by Palacpac et al. (1999), Bakker et al. (2006) and others pointed out one more challenge, namely how delicate the glycosylation system is – coexpression of the human β1,4-galactosyltransferase (β1,4-GalT) had led to the production of galactosylated, but also of unusual, hybrid-type glycans. The latter was due to activity of the galactosyltransferase at a suboptimal stage of the glycosylation pathway and interference with the endogenous glycosylation reactions (described in detail below). Similar findings were reported upon overexpression of *N*-acetylglucosaminyltransferase (GnT) III (Rouwendal et al., 2007; Frey et al., 2009; Karg et al., 2010; Castilho et al., 2011; detailed description see below). These examples show that the final glycosylation profile of a co-expression approach depends on various factors, including the availability of (i) acceptor glycan and (ii) donor substrate as well as (iii) the correct subcellular targeting of the recombinant glycosyltransferase in order to avoid interference with the endogenous glycosylation machinery.

## TARGETING MECHANISM OF GLYCOSYLTRANSFERASES

The glycosylation reactions within the Golgi are carried out in a sequential, stepwise manner, and one reaction can be the prerequisite for another one – or inhibit it. Therefore, the ordered sequential arrangement of enzymatic activities, i.e., the correct subcellular localization of the involved enzymes is of utmost importance. This tight regulation has consequences for the expression and targeting of heterologous glycosylation enzymes, as they need to fit precisely into the existing pathway. Fine-tuning the subcellular localization of heterologously expressed glycosylation enzymes requires vast knowledge of the underlying targeting mechanisms.

**FIGURE 2:**
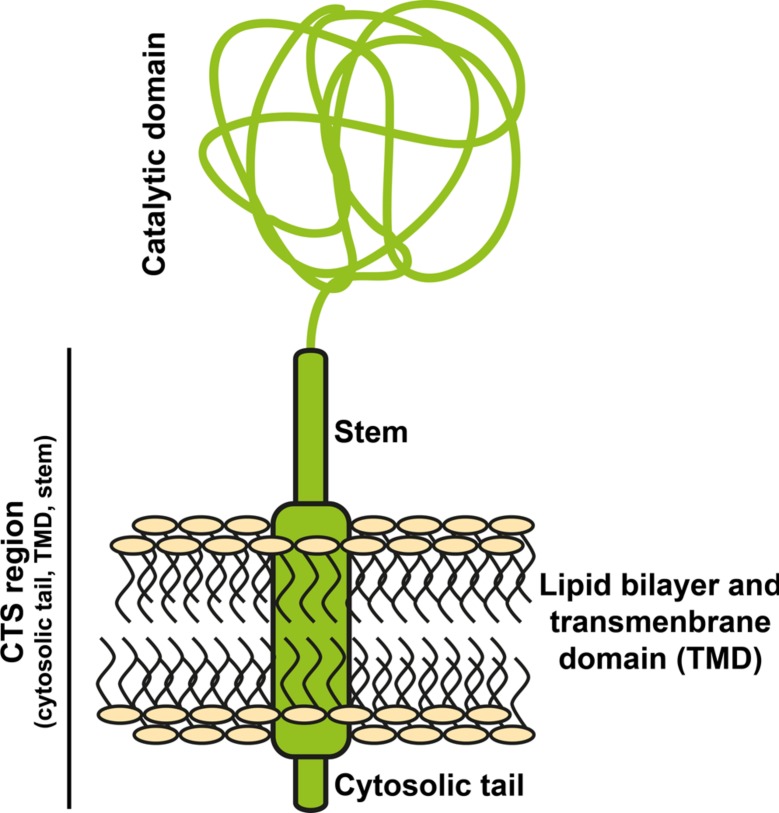
**Structure of *N-*glycosyltransferases**. Golgi-located glycosyltransferases are type II transmembrane proteins. Their localization within the Golgi depends on the N-terminal CTS region, consisting of the cytosolic tail, the transmembrane domain and a stem. The C-terminal catalytic domain is directed to the Golgi lumen.

All known Golgi-resident *N*-glycosyltransferases are type II transmembrane proteins (reviewed by, e.g., Schoberer and Strasser, 2011). Their N-terminus is exposed to the cytoplasm, followed by a transmembrane domain, a stem, and the catalytic domain (see **Figure 2**). The cytoplasmic part, transmembrane domain, and stem are referred to as CTS region and are responsible for targeting the enzyme to the correct compartment (Essl et al., 1999), and even sub-compartment. This was shown by different Golgi-localized glycosyltransferases not present within the same sub-compartment (Saint-Jore-Dupas et al., 2006; Schoberer et al., 2009, 2010).

Research activities to elucidate the targeting mechanism(s) of type II transmembrane proteins revealed a basic conservation of processes between plants and mammals (Schoberer et al., 2010). However, the question on how the fine-tuning of targeting works has not been answered definitively, but several factors have been identified. For example, the cytoplasmic tail influences ER export of the enzyme (Schoberer et al., 2009) and can change the final destination of a protein (Jiang and Rogers, 1998). In mammalian cells it has been shown to relocate the catalytic domain from one to another Golgi subcompartment (Uliana et al., 2006). This might be due to formation of homo- or heterodimers of the enzymes or interaction with other proteins (Schoberer et al., 2013). Also the length of the transmembrane domain might influence targeting (Pagny et al., 2003), as the membrane thickness of the Golgi changes from *cis* to *trans*. Finally, the composition of the lipid bilayer surrounding the enzyme might influence targeting, too (reviewed by Schoberer and Strasser, 2011). This makes the CTS regions of glycosyltransferases key regulators for precise sub-Golgi targeting. Theoretically, CTS domains from any eukaryotic organism may serve as a suitable targeting domain. Recent genome sequencing projects provided an abundance of such sequences that may be used for targeted sub-Golgi localization (Ohtsubo and Marth, 2006; Varki, 2006). Nevertheless, as the molecular mechanisms that lie behind this fine-tuning of targeting are not fully understood, it is not entirely predictable how CTS sequences actually perform when fused to the catalytic domain of another glycosyltransferase and expressed in a foreign cell. Thus, experimental testing is required. Also the prediction of the exact size of the individual glycosyltransferase-domains (cytosolic tail, transmembrane domain, stem, catalytic domain) is difficult and even though bioinformatics prediction technology has improved in recent years, different algorithms can lead to different results. Therefore, the reliability of the identification of the separate domains should be considered carefully.

## CHIMERIC GLYCOSYLTRANSFERASES USED FOR *IN PLANTA* GLYCOENGINEERING

**FIGURE 3:**
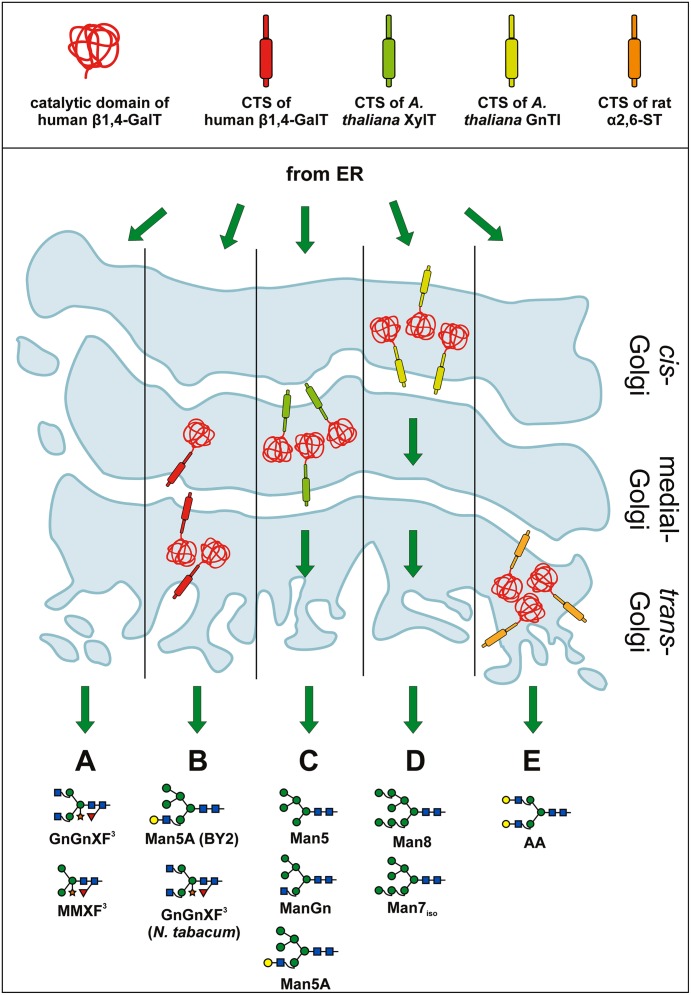
**Expression of β1,4-GalT and chimeric versions thereof in plants.** Schematic presentation of various β1,4-GalT constructs expressed in plants and the consequences on the glycosylation profile of total and recombinantly expressed proteins. In the top panel β1,4-GalT catalytic domain and various CTS regions are illustrated in different colors. The color code is used to better visualize various chimeric fusion constructs. The bottom panel shows a Golgi stack and the hypothetical localization of different β1,4-GalT constructs. Green arrows indicate cargo flow from ER through the Golgi. Major glycan structures produced under the given conditions are shown. **(A)** Major glycoforms detected in wild-type plants (without the expression of β1,4-GalT) are complex *N*-glycans carrying xylose and fucose (i.e., GnGnXF, MMXF, etc.; e.g., Bakker et al., 2001). **(B)** Expression of full-length human β1,4-GalT in BY2 tobacco cells (Palacpac et al., 1999) and tobacco plants (Bakker et al., 2001) led to different results. In BY2 cells, mainly galactosylated, hybrid-type glycans (like Man5A) as well as oligomannosidic glycans were found (Palacpac et al., 1999). In tobacco plants (Bakker et al., 2001) GnGnXF remained the major glycoform and only small amounts of galactosylated glycans were found. These results indicate that β1,4-GalT acted in BY2 cells at an earlier stage of the glycosylation pathway than in tobacco plants, leading to interference with endogenous glycosylation reactions in cells, but not in plants. **(C)** Major glycoforms detected upon expression of a chimeric GalT, that carries the CTS region of *A. thaliana* β1,2-xylosyltransferase (indicated in pale green) and targets the enzyme to a medial stage of the glycan processing pathway: Man5, ManGn, Man5A. A drastically reduced amount of xylosylated and fucosylated glycans was detected (Bakker et al., 2006). The results point to an early activity of the chimeric β1,4-GalT, most probably in medial Golgi stacks. **(D)** Targeting the GalT to an even earlier compartment by fusing it to the CTS of the *cis*-Golgi acting GnTI (indicated in yellow; Vézina et al., 2009) induced the production of nearly exclusively oligomannosidic structures. Only minute amounts of galactosylated, hybrid Man5A were present. **(E)** Upon expression in a XT/FT knock-down plant line of a chimeric GalT carrying the late-Golgi CTS of rat α2,6-sialyltransferase (indicated in orange) proteins carrying mainly galactosylated glycans (e.g., AA) were generated (Strasser et al., 2009). These results indicate that the ST-GalT fusion is indeed located in a late Golgi stack where final *N*-glycan processing takes place.

First steps to shift the plant *N*-glycosylation pattern from plant-specific *N*-glycans (i.e., GnGnXF, MMXF, etc.; see **Figure 3A**) toward the production of human-like structures were based on full-length mammalian glycosyltransferases. Expressing a human β1,4-GalT in tobacco plants (Bakker et al., 2001) and tobacco BY2 cells (Palacpac et al., 1999) in fact resulted in galactosylated glycans, however, other oligosaccharides were produced as well. In BY2 cells, unusual and incompletely processed glycans lacking xylose and fucose (e.g., Man5A) were abundant (see **Figure 3B**), indicating interference of the heterologously expressed GalT with endogenous glycan processing enzymes like mannosidase II, GnTII and plant-specific β1,2-xylosyltransferase and α1,3-fucosyltransferase (XT and FT). In tobacco plants, xylosylated, and fucosylated GnGnXF remained the main oligosaccharide (see **Figure 3B**) and only minor amounts of galactosylated oligosaccharides like AAXF or GnAXF were found. This points to activity of the GalT at a later stage in tobacco plants, after completion of the endogenous glycosylation reactions. At this later stage, interference with the endogenous glycosylation reactions did not take place and therefore the amount of β1,2-xylose and α1,3-fucose – residues unwanted on proteins needed for human applications – remained basically unchanged. In order to transfer the down-regulation effect observed in BY2 cells to tobacco plants and reduce/eliminate the two plant-specific glycan residues (β1,2-xylose and α1,3-fucose), a chimeric version of the human GalT was constructed that contained the CTS region of the *A. thaliana* β1,2-xylosyltransferase (Bakker et al., 2006). The intention was to generate a chimeric enzyme that acts in the medial Golgi, simultaneously or prior to the endogenously present β1,2-xylosyltransferase and core α1,3-fucosyltransferase (XT and FT). As β1,4-galactosylated proteins are no longer substrates for XT and FT (Staudacher et al., 1995; Kajiura et al., 2012), the expression in tobacco plants led to the intended, drastic decrease in plant-specific glycans. However, the early activity of the β1,4-GalT also led to the inhibition of other enzymes – like mannosidase II and GnTII – and thus to the generation of substantial amounts of unusual, incompletely processed glycans (oligomannosidic glycans, Man5A, etc.; see **Figure 3C**). Altogether, the glycan profile was similar to the profile of BY2 cells expressing the full-length human β1,4-GalT (Palacpac et al., 1999). This indicates that the chimeric construct in tobacco plants and the full-length, human GalT in BY2 cells show activity at a comparable stage of the glycosylation pathway.

Targeting the human β1,4-GalT to an even earlier compartment (ER/*cis*-Golgi) by fusion with the CTS region of the *A. thaliana* GnTI further increased the amount of oligomannosidic glycans (Vézina et al., 2009; see **Figure 3D**). Galactosylated oligosaccharides were hardly found, indicating increased interference with the glycosylation machinery and the secretory pathway.

With the advent of XT/FT knock-down or knock-out lines (Koprivova et al., 2004; Cox et al., 2006; Schähs et al., 2007; Strasser et al., 2008; Shin et al., 2011) a more elegant way to prevent plant-specific glycosylation had been established and the aim of co-expressing human GalT in plants shifted from “interfering with endogenous reactions” to “generating homogeneously galactosylated, human-type glycans.” This was achieved by a rationally designed construct targeting the GalT to a late Golgi compartment. Fusions of the catalytic GalT domain to the CTS region of α2,6-sialyltransferase, an enzyme acting in the final steps of the mammalian glycosylation pathway, indeed resulted in the generation of human-type, mono- and di-galactosylated glycans in XT/FT knock-down plants (Strasser et al., 2009; see **Figure 3E**). This was an important step for the *in planta* generation of proteins carrying fully human glycans.

This semi-synthetic approach was applied to GnTs to further explore the consequences of generating hybrid constructs carrying foreign CTS regions. One of the GnTs, β1,4-mannosyl-β1,4-*N*-acetylglucosaminyltransferase (GnTIII), catalyzes the formation of so-called bisected glycans (Carver et al., 1981; Narasimhan, 1982), a modification frequently found on human proteins but not present in plants (Rouwendal et al., 2007). Importantly, bisected – as well as the previously mentioned β1,4-galactosylated glycans – cannot be modified with plant-specific xylose or fucose residues (Rouwendal et al., 2007). In order to produce such bisected glycans and thus prevent the addition of plant-specific glyco-epitopes, fully human GnTIII and a hybrid construct (the catalytic domain was fused to the CTS region of *A. thaliana* α-mannosidase II) were expressed in tobacco plants and BY-2 tobacco cells. The chimeric constructs led to a stronger decrease in plant-specific glyco-epitopes most probably due to targeting to an early/medial Golgi subcompartment (Rouwendal et al., 2007; Frey et al., 2009; Karg et al., 2010). However, targeting of GnTIII to an early compartment not only inhibited unwanted reactions but also led to the generation of non-standard, mainly hybrid-type glycans. Transiently expressing a series of GnTIII-constructs containing different CTS regions (*A. thaliana* Golgi mannosidase II, *A. thaliana* core α1,3-fucosyltransferase, *A. thaliana* β1,2-xyloslytransferase, and rat α2,6-sialyltransferase) in XT/FT knock-down *N. benthamiana* plants identified late targeting sequences as preferential for the production of naturally occurring, bisected *N*-glycans (Castilho et al., 2011).

In a similar approach, enzymes responsible for branched glycans (i.e., tri- and tetraantennary glycans; human α1,3-mannosyl-β1,4-*N*-acetylglucosaminyltransferase IVa (GnTIV) and human α1,6-mannosyl-β1,6-*N*-acetylglucosaminyltransferase V (GnTV)) were tested with different CTS regions (endogenous CTS region, rat α2,6-sialyltransferase, *A. thaliana* core α1,3-fucosyltransferase, *A. thaliana* β1,2-xyloslytransferase; Castilho et al., 2011; Nagels et al., 2011, 2012a,b). The fusions with the medial Golgi-targeting CTS region of, e.g., FT allowed the generation of a high degree of branched glycans on co-expressed reporter proteins, whereas the endogenous CTS and the late Golgi CTS from α2,6-sialyltransferase did not lead to substantial amounts of branched glycans.

Collectively these results demonstrate that it is not sufficient to “simply” introduce a foreign glycosylation enzyme into a plant to obtain a desired glycan structure. Instead, the successful production of proteins with human-type *N*-glycosylation in plants harbors a large number of challenges and requires knowledge of glycosylation pathways, enzyme specificities and related topics, like subcellular protein transport. Semi-synthetic approaches serve as useful tools to approach these challenges.

## FURTHER CHALLENGES

In recent years, a variety of expression hosts were glyco-engineered (recently reviewed by, e.g., Jacobs and Callewaert, 2009; Loos and Steinkellner, 2012) and the first products with enhanced properties in animal studies have reached the clinic (Ratner, 2014). Plants, with their similar-to-human yet more simple *N*-glycosylation machinery and their amenability to glyco-engineering have been on the forefront of this development. Despite substantial achievements, the advantages of this system have been used only in a few *in vivo* studies (Bendandi et al., 2010; Forthal et al., 2010; Zeitlin et al., 2011, 2013; Hiatt et al., 2014). So far only one plant-produced product has reached the market, i.e., glucocerebrosidase to treat Gaucher’s disease. This protein carries terminal mannose residues, a glycosylation form that confers enhanced efficacies (Grabowski et al., 2014). Another plant-produced glyco-optimized protein drug has recently been used to treat patients: ZMapp, an experimental mAb cocktail against Ebola virus was given to several individuals during the ongoing Ebola epidemic (critically reviewed by Goodman, 2014). These antibodies had not yet gone through clinical studies but due to the dire predictions of Ebola virus infection and lack of other treatment options its application had been approved by regulatory authorities in several countries.

With the appearance of efficient, transient expression methods, the rapid, scalable and cost-effective production of high-value recombinant proteins became possible (Gleba et al., 2014). However, to realize the full potential of plant biotechnology, advanced, stably glyco-engineered plant strains in combination with semi-synthetic approaches will be needed. Versatile, modular expression vectors like MoClo (Weber et al., 2011) and GoldenBraid (Sarrion-Perdigones et al., 2013) allow efficient shuffling of domains and will certainly speed up the generation of constructs. Quick assembly of multi-gene vectors also simplifies the remodeling of glycosylation pathways as recently demonstrated (Schneider et al., 2014). Moreover, new technologies for genome editing, like CRISPR and TALENs (Lozano-Juste and Cutler, 2014) allow efficient elimination of genes and facilitate metabolic engineering and reprogramming of biosynthetic processes. These developments in combination with computer modeling and simulation approaches that predict protein–glycan interactions will accelerate the development of drugs with optimized and even new functions. In sum, the currently available gene expression systems and the new tools offered by synthetic biology create an ideal environment for establishing a plant-based biomanufacturing platform that can compete with or even surpass current industry standards.

## Conflict of Interest Statement

The authors declare that the research was conducted in the absence of any commercial or financial relationships that could be construed as a potential conflict of interest.
